# Supplementary material for: Linear reinforcement learning in planning, grid fields, and cognitive control
Source: Nat Commun. 2021 Aug 16;12:4942. doi: 10.1038/s41467-021-25123-3 (PMC8368103; doi:10.1038/s41467-021-25123-3)
Supplement: Supplementary file 1 — Supplementary Information [file 41467_2021_25123_MOESM1_ESM.pdf]

Supplementary Information

# Linear reinforcement learning in planning, grid fields, and cognitive control

Payam Piray[1,*] and Nathaniel D. Daw[1]
Princeton Neuroscience Institute, Princeton University, Princeton, NJ, USA.
*Corresponding author: ppiray@princeton.edu

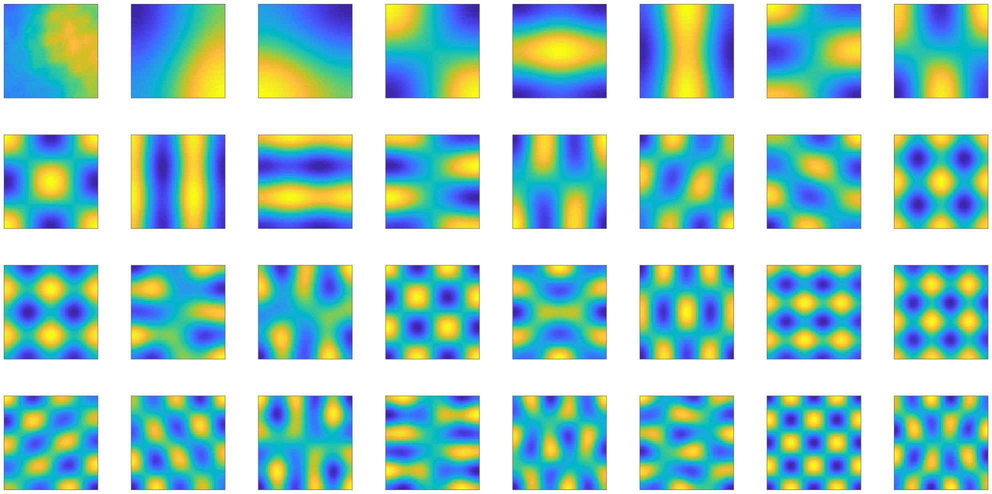

Supplementary Fig 1. The first 32 eigenvectors (corresponding to the 32 largest eigenvalues) of the DR from a 50-by-50 maze are plotted. All state costs are assumed to be 0.1.

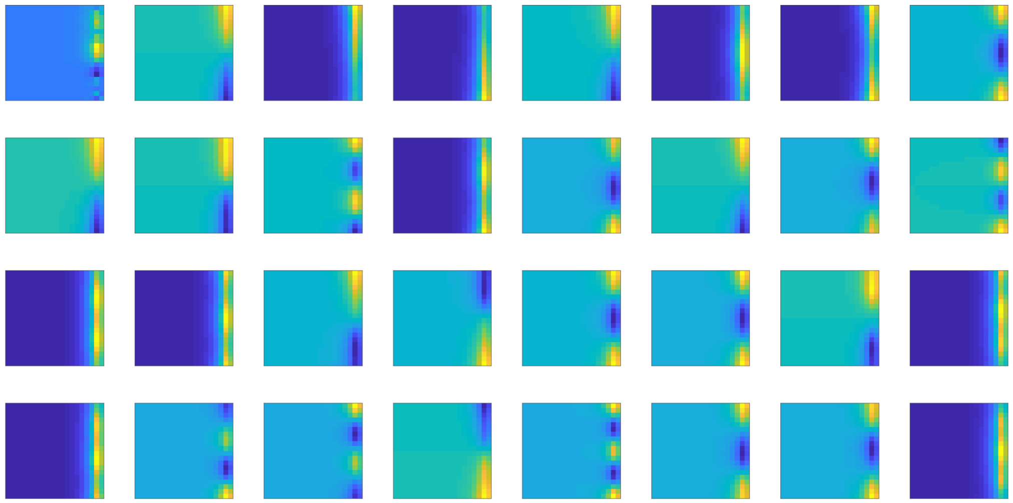

Supplementary Fig 2. Different border cells corresponding to the largest 32 eigenvectors in a 20-by-20 maze.

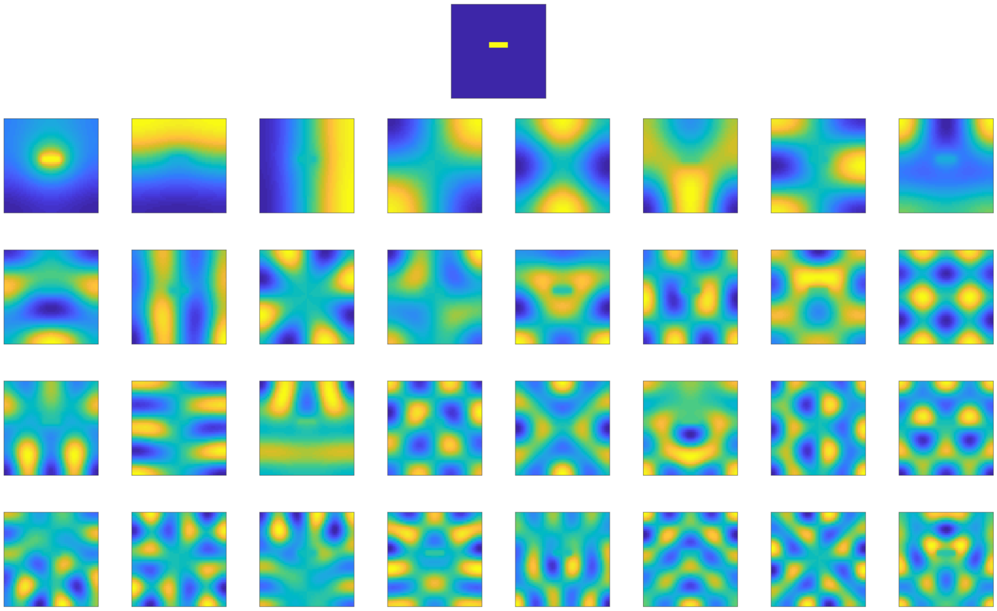

Supplementary Fig 3. Effects of state costs on eigenvectors of the DR in a 50-by-50 maze. Top: cost across all states. The cost is 0.1 for all states except those yellow states in the middle of the maze in which the cost is 0.5. Other plots show the top 32 eigenvectors of the DR.
